# *From goods to goats*: examining post-disaster livelihood recovery in the aftermath of the Nepal earthquake 2015

**DOI:** 10.1007/s11069-022-05543-0

**Published:** 2022-08-18

**Authors:** Jeevan Karki, Steve Matthewman, Jesse Hession Grayman

**Affiliations:** 1 Department of Sociology, University of Auckland, Auckland, New Zealand; 2 Department of Development Studies, University of Auckland, Auckland, New Zealand

**Keywords:** Aid, Nepal earthquake, Post-disaster recovery, Rural livelihoods, Social reproduction

## Abstract

Disasters can have substantial impacts on people’s livelihoods in developing countries. Further, if the need for livelihood interventions is ignored or delayed, the crisis may trigger unexpected harmful consequences in the affected households in the aftermath. Therefore, restoring livelihoods should remain a priority in the post-disaster recovery process. However, such recoveries in rural contexts and developing countries, like Nepal, are complex as the livelihood restoration process is affected by serious spatial, socio-economic, and political factors. We employed qualitative research methods in four highly affected districts in the 2015 Nepal Earthquake (7.8 M_w_) to examine post-disaster livelihoods recovery. Our paper critically assesses the humanitarian response based on the narratives and lived experiences of affected households. The findings show that humanitarian assistance was crucial in addressing several unmet needs of disaster-affected rural households in resource-poor settings in Nepal. However, the interventions were generally fragmented, insufficient, neoliberal led (forcing market dependencies), and largely business-as-usual in their orientation. Previous studies in Nepal paid insufficient attention to the goods provided to affected households in the name of recovery. Therefore, our paper scrutinises selected humanitarian objects, such as power tillers, and unpacks their political economy and effectiveness in local contexts. Further, our findings show that some livelihood policies reinforced the gap between the haves and have-nots, thereby reproducing pre-disaster inequalities in the post-disaster field.

## Introduction

Disasters can have a significant impact on rural livelihoods. They can inflict substantial damage on farmland or resources (natural or physical) on which people depend for their subsistence or income (Epstein et al. 2018; Lebel et al. 2006). Disasters can kill or injure livestock that are valuable assets in developing countries, disrupt markets and supply chains, and damage lifeline utilities (such as electricity or telecommunications), all of which are essential for farming or micro-entrepreneurial activities (Daly et al. 2020; Epstein et al. 2018). People adapt to their post-disaster everyday life using different Indigenous or local coping strategies in the short-run (see Gaillard et al. 2009); however, delays or ineffective recoveries may have several unexpected socio-economic consequences for disaster-affected households (ADPC 2015, p. 10). For example, poor parents may decide to take their children out from school due to a lack of money. These out-of-school children are likely to be recruited into child labour (Sassi 2021; UNOCHA 2016). Similarly, people may sell their assets making them further vulnerable, and cases of girls’ trafficking or forced/unsafe labour migration might increase in the aftermath of disasters or acute crises (Bishokarma 2012; UNOCHA 2021). Therefore, it is crucial to help the most disadvantaged households in the communities restore their livelihoods as early and effectively as possible.

However, restoring livelihoods in developing countries, like Nepal, and in rural contexts are quite complex. The recovery process is affected by several factors such as place-based determinants (geographies that shape everyday economies such as rural tourism or farming; market-centre or hinterland), the informal nature of the economy, the diverse needs of disaster survivors, and intersectional issues (such as caste, ethnicity, and gender). Further, poverty is generally pervasive and embedded in rural households. Rural people generally lack the necessary assets and financial resources to restore their livelihoods in the aftermath of disaster. Moreover, insurance for micro-enterprises, livestock or crops, and assets is rare in developing countries (De Mel et al. 2012; Devkota et al. 2021) which makes post-disaster livelihood restoration even more challenging. Aid or external assistance plays a vital role at this juncture to help disaster survivors restore livelihoods following calamity (Coate et al. 2006; Daly et al. 2020; Khan et al. 2015).

Nepal experienced a powerful earthquake (7.8 M_w_) in April 2015 that claimed nearly 9000 lives, injured more than 20,000 people, and disrupted the livelihoods of millions of households (NPC/GoN 2015). Rural people lost their livestock, planting seeds, and agricultural tools. The condition of grazing and crop fields eroded in several communities after the earthquake, and agricultural inputs and services (such as irrigation) were unavailable, damaged or inaccessible. According to the Post-Disaster Needs Assessment (PDNA) report prepared by the National Planning Commission of Nepal Government (NPC/GoN 2015, p. 215), the earthquakes impacted the livelihoods of about 2.29 million households. Similarly, the PDNA estimated that over 17,000 cattle and about 40,000 smaller domesticated animals perished. Additionally, the earthquake damaged irrigation facilities and triggered landslides in different locations, rendering nearly 1000 hectares of land useless, as documented by the report.

In the earthquake-affected districts of Nepal, several government institutions and humanitarian/aid agencies remained active, seeking to restore the livelihoods of impacted households. However, empirical and critical studies on these recovery interventions and on people’s lived-experience, particularly among poor and marginalised groups, are scant. Therefore, our study aims to analyse livelihood recovery interventions and disaster survivors’ lived-experience in the four most severely affected districts in Nepal, viz.—Dhading, Gorkha, Rasuwa and Sindhupalchok.

Our paper critically underscores the humanitarian response based on the narratives and lived-experience of affected households. Our findings show that recovery programmes from different stakeholders were necessary, given the aforementioned earthquake impacts, widespread poverty, and social inequality. Further, these interventions were shown to provide essential help that addressed several unmet needs in disaster-affected households. However, these interventions could be improved. They were generally fragmented, insufficient, neoliberal in orientation (prioritising market solutions), proffered business-as-usual practices, and consequently, the enterprise/entrepreneur-related interventions failed to benefit the poorest and most marginalised households in these communities.

## Conceptualising livelihood recovery

**Fig. 1﻿:**
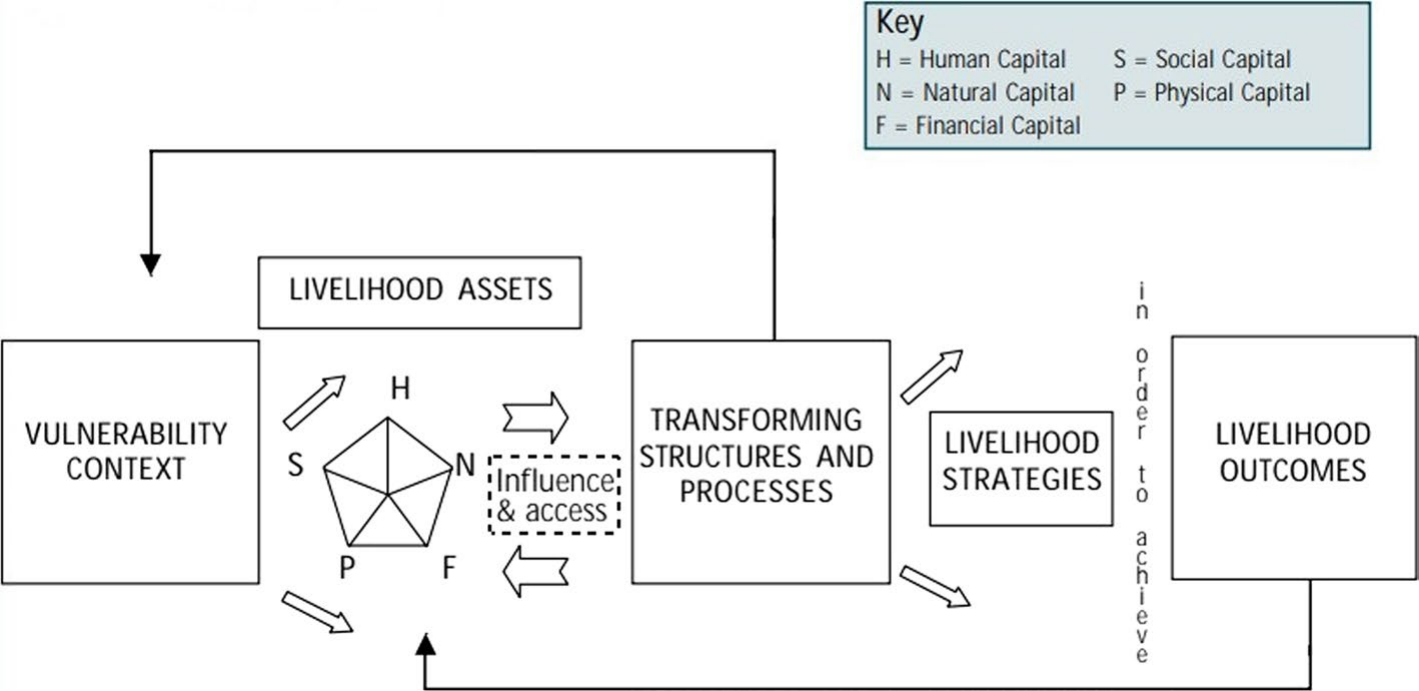
Sustainable Livelihoods Framework. *Source*: DFID (2001, p. 1.1)

Various definitions of livelihoods or sustainable livelihoods are offered in the literature; however, a simple and comprehensive definition is provided by Robert Chambers and Gordon R. Conway, who are influential thinkers in the field of development. Based on their work, the Department for International Development (DFID) conceptualised the sustainable livelihoods (SL) framework (see Fig. 1) which has been widely used in the aid and development sector. DFID (2001, p. 1.1) defines livelihood and sustainable livelihoods as follows:

A livelihood comprises the capabilities, assets (including both material and social resources) and activities required for a means of living. A livelihood is sustainable when it can cope with and recover from stresses and shocks and maintain or enhance its capabilities and assets both now and in the future while not undermining the natural resource base.

The concept of capitals/assets (or the asset pentagon, viz. human, natural, financial, social and physical capital) lies at the heart of the SL framework (see DFID 2001). The framework encourages exploring opportunities for strengthening, interchanging, exchanging or substituting different forms of capital. The SL approach can also be appreciated for putting people at the centre of development, considering the circumstances of their vulnerability, and taking a holistic approach to improving people’s livelihood and wellbeing. However, some scholars are critical of the livelihoods approach for lacking appraisal of (or not being explicit about) power and politics in its framework while designing and implementing community development or disaster recovery programmes (Kapadia 2014; Scoones 2009).

In addition to the SL framework, social vulnerability, resilience, and social capital–which have become dominant concepts in disaster scholarship–provide a helpful supplementary framework for understanding the impacts on, and recoveries of, livelihoods following disasters. Wisner et al. (2004, p. 11) define vulnerability as “the characteristics of a person or group and their situation that influence their capacity to anticipate, cope with, resist and recover from the impact of natural hazards” (Italics in original). The social vulnerability literature shows that some groups in society are more exposed than others to disasters and, therefore, more likely to suffer from their impacts (Wisner et al. 2004; Hewitt 1997). Thus, disasters are seen to exacerbate already existing inequalities. The disadvantaged and marginalised households in the communities are likely to have limited resources to safeguard their livelihood assets and may have fewer financial resources and opportunities to return to the status quo ante (Tierney 2019; Wisner et al. 2004). However, vulnerability scholarship can pathologise people and overlook their agency and adaptive coping capacities which they can use in the disaster recovery process (Hewitt 1997). Indeed, having already lived in miserable conditions, they may have developed *everyday resilience* to cope with such hardships, providing resources to draw on during disaster (see Uekusa and Matthewman 2017).

Similarly, the concept of resilience has also become a mantra in recent years in the aid and development sector, and is central to debates within the field (Levine et al. 2012; Manyena et al. 2011). It gained popularity due to its strengths-based conception in contradistinction to the concept of vulnerability. While various definitions have been offered by different scholars and organisations, we use the following in this paper (DFID 2011, p. 6):

Disaster Resilience is the ability of countries, communities and households to manage change, by maintaining or transforming living standards in the face of shocks or stresses–such as earthquakes, drought or violent conflict–without compromising their long-term prospects.

Resilience has been accepted by key international policies for Disaster Risk Reduction and development, such as Sendai Framework and Sustainable Development Goals. The etymological meaning of resilience is *‘to jump back’,* derived from the Latin word *resilio* (Klein et al. 2003, p. 35) or *resiliere* (Sudmeier-Riex 2014, p. 69). Resilience is, therefore, commonly viewed as the ability to “absorb the shocks” and “bounce back” from disasters. Nevertheless, resilience is not free from criticism. First of all, it has become a buzzword in the humanitarian-development sector (see Alexander 2013). The strongest criticism of resilience discourse is that it is aligned with the neoliberal project. In this regard, the idea of resilience is orchestrated to deflect the state’s responsibilities to its citizens. Instead, victims are ‘responsibilised’. They must prepare for disasters, manage post-disaster trauma, and recover on their own (Davoudi 2018; Tierney 2015), often absent adequate resourcing.

As with resilience thinking, the role of social capital in disaster recovery has received much attention in recent years (Uekusa et al. 2022). Social capital is generally defined as social trust, networks, and relationships that people can draw upon. Several studies have revealed that families and communities having high social capital are more likely to bounce back after disasters (Akbar and Aldrich 2018; Aldrich 2011; Bhandari 2014; Nakagawa and Shaw 2004). These studies have shown that such communities with strong social connections and high levels of trust are likely to exchange knowledge, skills, finances or material resources (including labour) for rebuilding and recovery efforts and provide emotional support to cope with the stress. Hence, social capital is considered a crucial component of disaster resilience. However, many scholars (see, for example, Bankoff 2015; Roberts 2019) have noted that social capital alone is insufficient to make a family or neighbourhood resilient. Moreover, social capital is also predicated on exclusions: only some people are able to connect, only some are allowed to belong, and only some are trusted.

## Rethinking livelihoods and recovery in developing countries

Livelihoods in developing countries are generally informal, subsistence-based, and farming or agro-entrepreneurship centred. Therefore, a high proportion of the population in developing countries engage in the informal sector economy, predominantly in agriculture, including livestock and fisheries (see Coate et al. 2006; Daly et al. 2020; NPC/GoN 2015; Thorburn 2009). Further, their livelihood strategies are diversified (see Chatterjee and Okazaki 2018; Chhotray and Few 2012; Daly et al. 2020). Therefore, people in developing countries have multiple sources of livelihood which they live on, such as agriculture, small/micro-enterprises, wage labour or employment (temporary or permanent), and remittances (Eadie et al. 2020; He et al. 2018).

Historically, agriculture was a male-dominant sector in many parts of the world, whereby the major decisions and key tasks were taken or assigned by male members. However, this pattern seems to be gradually changing over the decades in many parts of the world. Scholars have referred to this phenomenon as the ‘feminisation of agriculture’ (Tamang et al. 2014; Zhllima et al. 2021). In many countries, this paradigm shift is due to the absence of male members in the family because of labour migration (Pandey 2021; Tamang et al. 2014). Also, adult male members in the households tend to leave their families if a conflict or armed insurgency breaks out at the local level or in the region (Menon and Rodgers 2015). In this connection, most young or adult male members left their village during the Maoist insurgency in Nepal to work, study or live in cities. Many of them left the country itself to work as labour migrants in Malaysia or the Gulf states. The young men in the conflict-affected villages developed a fear that they might be recruited by Maoist insurgents or suspected by the state to be associated with Maoist militias. Furthermore, the increased involvement of female members in economic activity is also due to the policies and strategies of government and/or non-government organisations (NGOs) which tend to focus on women as beneficiaries of their income-generating programmes.

Marginal farmers’ lives are usually most affected by disasters as their livelihood activities are generally unprotected, they have limited options or alternatives to restore/revitalise them, and they lack capital for reinvestment (Lebel et al. 2006). Physical or economic livelihood assets are few or scarce in poor and disadvantaged households. Therefore, many of them take the risk of protecting livelihood goods or assets at the expense of their own lives (see Eadie et al. 2020). For example, instead of evacuating during a disaster, some family members might stay back to protect assets such as livestock or stored food grains.

After a catastrophe, livelihood recovery is often initiated with aspirations to build back better and strengthen resilience; however, many cases around the world reveal that such slogans tend to fade away quickly, and survivors return to their previous state of vulnerability due to the status quo or business-as-usual phenomenon (see Chhotray and Few 2012). In this regard, Chhotray and Few argue that repetitive or recurring hazard contexts, poor institutional support, weak grassroots adaptive capacity, and a lack of sustained support are the main reasons for the lack of transformative changes to livelihoods even long after the calamity has taken place.

State and non-state actors, particularly aid organisations, are key players in many developing countries for helping people restore their livelihoods following disasters (see Coate et al. 2006; Daly et al. 2020). Nevertheless, researchers have continuously suggested that improving post-disaster livelihoods recovery assistance has several problems. One common problem is the duplication of resources. Following Typhoon Yolanda in the Philippines, the same household received fishing boats from multiple humanitarian organisations, whereas some other families did not receive anything at all (Eadie et al. 2020). Further, Chhotray and Few (2012) and Daly et al. (2020) argue that rather than piecemeal or fragmented interventions, a sustained effort is necessary to help the most vulnerable disaster-affected households restore their livelihoods effectively and sustainably.

Finally, housing reconstruction and livelihoods are strongly interlinked. Therefore, housing reconstruction and livelihoods recovery should be integrated because livestock management and harvest storage are associated with rural life in Nepal, and domestic space may be used for income-generating activities; however, such an approach failed in Nepal’s reconstruction and recovery process (see Karki et al. 2022a). Overall, the housing reconstruction programme has drawn criticisms for undermining people’s voices and participation in the planning and decision-making process, and for replacing vernacular design with concrete houses that are spatially insufficient for family members, climatically unsuitable for their location, and practically inconvenient for everyday rural life (see Karki et al. 2022a).

## Study locations and methodology

### Research contexts, locations and participants

The research interest grew out of Author1’s first-hand experience of the 2015 Nepal Earthquake. After experiencing this devastation and loss, he was motivated to understand the lived experiences of the poorest and most marginalised peoples, the earthquakes’ impacts on them, and their recovery processes. Therefore, this paper is part of a larger research project that analyses post-disaster recovery and redevelopment among poor and marginalised social groups in the aftermath of the 2015 Nepal Earthquake. The research was carried out five years after the earthquake. This timeframe is important for examining mid-to-long-term livelihoods recovery (and housing reconstruction) following the disaster. However, it should be noted that data collection was conducted during another disaster, this time a global pandemic (Covid-19). The pandemic disrupted the livelihood journeys and plans that were underway (which we will discuss in Sect. 5.6). And for the researchers, it meant that the original research methodology had to be revised due to the travel restrictions and disruptions that Covid-19 caused.

**Fig. 2:**
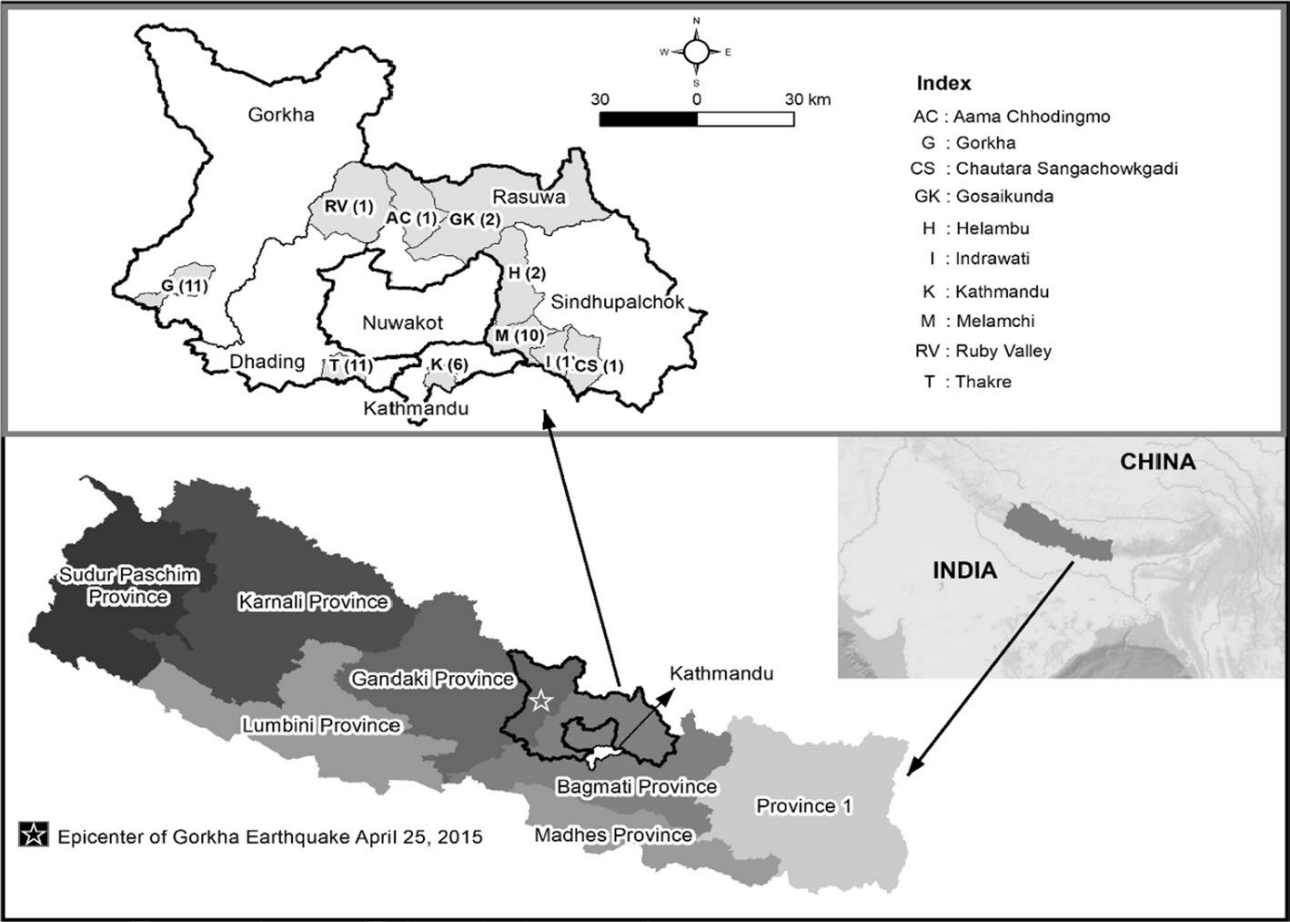
Map of Nepal showing research districts in the top inset. The figure in the parenthesis denotes the number of people interviewed from those regions. The map and its boundaries are for illustrative purpose only

The research was primarily carried out in Dhading, Gorkha (which was the epicentre of the 2015 Nepal Earthquake), Rasuwa, and Sindhupalchok Districts (see Fig. 2). These geographical locations located on the hills or at the foot of the mountains of central Nepal were among the worst-hit districts. The disaster survivors/community members and local NGO humanitarian/development workers were from these four districts. Government officials and some aid workers were based in Kathmandu, the capital city. A brief description of the four research districts is provided in Table 1 below.

**Table 1:** Situation of human development and the impact of the 2015 Nepal earthquake on human casualties and injuries and private/residential housing in the research districts

District	State of human development^a^			Casualties and damage due to the 2015 Nepal earthquake^b^		
	Human development index (HDI)	Life expectancy (years)	Adult literary (%)	Death	Injuries	Houses fully damaged
Dhading	0.461	70.86	53.26	680	1218	81,313
	*(0.490)*	*(68.80)*	*(59.57)*			
Gorkha	0.481	71.7	58.17	450	952	68,537
Rasuwa	0.461	70.91	41.32	681	771	11,950
Sindhupalchok	0.455	69.57	49.51	3570	1569	89,884

*Source*: ^a^National Planning Commission (https://www.npc.gov.np/human_development_indicators_by_district/). The figure in the parenthesis indicates the national average

^b^The Government of Nepal’s DRR portal (http://drrportal.gov.np/)

The research participants were purposefully selected from disadvantaged and marginalised social groups, namely Dalit and Adibāsi Janajāti peoples. The literal translation of Dalit is oppressed or downtrodden. Dalit people are socially and economically oppressed in Nepal. They face caste-based discriminatory practices in both public and private spheres. Similarly, Adibāsi Janajāti is an umbrella term to refer to ethnic groups in Nepal who have a distinctive collective identity of language, custom, and culture. The Chepang and Majhi, who participated in this research, are among the most disadvantaged and marginalised ethnic groups in the country. The government of Nepal has also recognised the current state of marginalisation and exclusion of different social groups from the mainstream of development (“Indigenous nationalities of Nepal…”, n.d.).

Both Dalit and Adibāsi Janajāti peoples have experienced systemic marginalisation and social exclusion since the unification of greater Nepal (see Gurung 2006; Hachhethu 2003; Tamang 2011). As a result, the highest rate of chronic and structural poverty today is found among Dalits and Adibāsi Janajātis (Wagle 2017). Moreover, Dalit people, particularly Tarāi Dalits, have the country’s lowest human development index (UNDP 2014). Therefore, the paper has identified both Dalit and Adibāsi Janajāti as marginalised groups due to their *shared history of marginalisation*, *disadvantaged positions* in society and *unequal development outcomes*.

Forty-six qualitative interviews were conducted with local people, humanitarian, and development workers in international and national non-government organisations (I/NGOs) and government representatives. Thirty-five of them were community people/disaster survivors, eight were humanitarian and development workers based in the research districts and Kathmandu (the capital city), and three were government representatives responsible for post-disaster recovery and reconstruction. The details of the research participants are detailed in Table 2.

**Table 2:** Types of research participants

Types of research participants	Female	Male	Total
(a) Disaster survivors (community members)	13	22	35
(b) Humanitarian/development workers	1	7	8
(c) Government representatives	–	3	3
Total	14	32	46

Further, Fig. 2 below depicts the research districts and *Gaunpalika/Nagarpalika* (rural municipality/municipality) and the number of research participants from those locations.

### Methods for data collection and analysis

The research followed qualitative research method principles. A relatively small number of cases were studied in-depth in order to explore the complexity of their situations and explain their “webs of meaning” (Merriam 2002; Ten Have 2004; Yin 2011). The research utilised multiple approaches to collect data from June 2020 to April 2021. Online and remote qualitative interviews were collected through digital technology such as Zoom, and also via telephone. Interviews in remote locations were undertaken by field assistants. The interviews were limited to one-off events due to the unprecedented challenges posed by the Covid-19 pandemic for travelling (international and local restrictions) and meeting (social distancing). Further, public angst was sensed or reported due to the overwhelming experiences of the unexpected pandemic.

The research asked the disaster survivors how the earthquake impacted their means of living and how they managed or sustained their livelihoods afterwards. We explored what kinds of livelihood assistance they received from government and non-government organisations and how such support was helpful (or not). The participants were also asked to compare and contrast their livelihood situation (pre- and post-earthquake) and opportunities in relation to other castes in the communities. Similarly, with government and humanitarian/development NGOs, we sought to understand the nature and rationale of livelihoods recovery interventions and the process of beneficiaries’ selection and project implementation in the communities. Interviewing both parties allowed us to understand their respective perceptions of the livelihood recovery interventions and their effectiveness.

The participants’ interviews were audio-recorded and transcribed. The transcripts were then transferred to NVivo software. We used an open coding process that included a close reading of the transcripts. Then, we grouped the open codes into categories and themes. After that meanings were extracted based on the identified themes.

Institutional ethics approval for the research was obtained from the researchers’ university.

## Study findings/results

### Types/typology of post-disaster recovery assistance

Table 3 below summarises a variety of post-earthquake recovery interventions undertaken by various I/NGOs and state agencies in the research districts. The table reveals that the livelihood assistance programmes included a wide range of activities: goods or material support, a goat support scheme, cash assistance, and skills/capacity enhancement.

**Table 3:** Post-disaster recovery assistance examples

Sector	Examples of recovery assistance
(d) Farming	Vegetable seed distribution, irrigation maintenance assistance, cash crop production, plastic tunnel support for commercial vegetable farming, hand-tractor (mini-tillers), beekeeping training, organic farming, high-value medicinal plants introduction, modern bee-hives support, paddy seeds, and maize corn seeds
(e) Livestock	Goat distribution, buck/billy goats (for mating or breeding purpose), financial assistance for livestock rearing, goat/cattle sheds management training, and pastureland improvement training
(f) Cash/voucher distribution	Cash/voucher assistance in exchange for labour/work undertaken for the community infrastructure recovery, such as rural trail repair and irrigation facility maintenance. Or cash assistance for post-disaster needs or livelihood recovery
(g) Tourism	Trekking guide training, hotel management training, food hygiene and accommodation sanitation training, and assistance to replace the lost or damaged tourist information signage
(h) Skills development/Capacity building	Skilled mason training, cell phone repair training, motorbike maintenance training, beautician training, and tailoring

*Source*: Based on the interviews with local NGOs and disaster survivors. This list is indicative rather than exhaustive

Although most of the disaster survivors reported that they received some of the above-mentioned items of assistance, fully one-third of our research participants reported receiving no livelihood or recovery-related assistance except for the government’s grant for housing reconstruction that was available to those who lost their homes in the earthquake.

While there were some overlaps between government and non-government assistance with livelihood recovery, most of the above-listed interventions were carried out by I/NGOs. The government officials interviewed in the National Reconstruction Authority revealed that their priority was on achieving housing reconstruction targets. Livelihood activities were therefore eclipsed. However, other relevant government authorities, such as the agriculture development office and the livestock development office, extended their programmes to support disaster survivors by providing seeds, livestock (such as goats), farming tools and technologies. Even so, the assistance seems to have been limited or one-off. Some participants articulated their frustration at failing to receive any assistance despite their frequent requests (to the officials) or at receiving a minimal amount of seeds.

Apart from the *goods* (materials) or *goats* (livestock) assistance, some organisations also disbursed cash. In this regard, an NGO in the Sindhupalchok District distributed NRP 25,000 (USD 250) for 2500 households in the selected eight Village Development Committees as part of their non-conditional cash support scheme for recovery.^1^ Similarly, an NGO in the Gorkha District also carried out a cash-for-work programme. In this scheme, the disaster-affected households had to renovate village paths, repair small irrigation facilities, repair damaged suspension bridges/crossings, clear roads after landslides, or undertake similar repair and maintenance tasks after the earthquake. Then, each household was entitled to work for nine or ten days. In doing so, the villagers were provided money based on the local daily wage rate. A few weeks after the earthquake, the local shops or market started to become operational again, and people needed cash to buy household needs in the market.

### Targeting/selection processes

The humanitarian NGO workers shared that they were advised by the government authorities to use the equal assistance principle (which was locally known or popularised as the *blanket approach*) while extending livelihood assistance to the community members. In this regard, Kalyan KC,^2^ an I/NGO worker in the Gorkha District, said his organisation wanted to support the affected households who were most in need and vulnerable. Vulnerable populations were identified by considering gender, age, disability, and caste/ethnicity. However, the NGO could not implement the targeted interventions as the government favoured a general approach. Kalyan stated:

We wanted to assist the targeted vulnerable peoples selected based on our selection criteria. However, the local government allocated a specific ward (geographical area) for us to work with and advised us to implement programmes intensively in that specified area. Therefore, we distributed seeds to everyone in that ward through our livelihood programme.

However, as Kalyan says, the NGO could not sustain this approach as financial resources were limited. Therefore, Kalyan and his team implemented other livelihood activities (in addition to seed distribution) with targeted households. This also shows that the government authorities did not strictly control, implement or monitor their own blanket approach.

Some other I/NGOs asked disaster survivors to demonstrate prior experience of undertaking entrepreneurship or micro-enterprises before they received assistance. As a result, relatively well-off people in the communities benefitted more from the entrepreneurship interventions (see Sect. 5.4 for further discussion on this topic). Similarly, an independent project evaluation indicated that some beneficiaries were established farmers in a livelihood recovery project (Christian Aid 2018).

### Farming interventions

Some NGOs had specific activities for households and livelihood recovery, such as entrepreneurship development; however, most NGOs engaged in a wide range of farming activities. Kalyan stated that his organisation considered several on-farm-related interventions based on the needs of the local communities. He said:

Some communities we work with live close to the market centres; therefore, we decided to help them with commercial vegetable farming. We distributed plastics for tunnel farming. We provided knowledge and techniques [for commercial farming] … Moreover, we also helped the communities with cash crops, such as providing citrus saplings. For others, we provided bee-hives. Oh yes, those who wanted to rear goats, we provided goats as well as training [on goat farming]. We also distributed maise seeds and soybean seeds.

These interventions were also confirmed in the interviews with the communities’ disaster-affected households. Biru (53), a male Chepang ethnic minority in the Dhading District, said, “…we were given vegetable seeds [to grow in our garden]. There were seeds of cauliflower and many other different kinds of vegetables. We grew them and ate. We were also able to sell some”. Sarala (62), a female Dalit from the same district, had a similar experience. She stated:

We did not have money to buy anything. From the agriculture office, we got some seeds. We planted them, looked after them and ate the vegetables when they were ready. The staff from the agriculture office came and saw it [our vegetable garden], and they encouraged us to continue.

The vegetable and crop seeds that people had stored in their homes were often buried under rubble caused by the earthquake, and it was expected that farmers may lack the money to buy new seeds or that the seeds may not be available in local markets (NPC/GoN 2015, p. 85). Therefore, seeds assistance was a reasonable response from the government and non-government humanitarian organisations.

However, Sagar (33), a male Majhi ethnic minority in the Sindhupalchok District shared that often the assistance was so meagre and insufficient. He said, “…one organisation comes and gives little seeds. They come and distribute 10 grams or 5 grams and advise us to sow them. That’s all. Then, they organise a few [community] meetings. This does not help! He added, “…everyone says ‘sipmulak- sipmulak’ (skills-based, skills-based), but I found them not that useful. And, they give you just a goat [for generating incomes].”

Some participants noted the unintended consequences of aid, stating that the assistance invited new forms of problems. Phul Maya (41), a female Chepang ethnic minority in Dhading, who had received a hand-tiller shared her experience:

We were given hand-tiller [for ploughing our land]. But if something happens to it, I mean, if the tiller breaks down, it will be challenging to repair. The repair centre is also far away to take it there for repair and bring back when it is done. The tiller thing turns out to be more complex than the oxen plough we used to have.

### Entrepreneurship for recovery?

One aid organisation supported specific disaster-affected individuals in the Rasuwa District for entrepreneurial development. One of the selection criteria for participants was that the people needed to have previous experience of undertaking small business or entrepreneurship-related activities. At a bare minimum, the aid organisation wanted someone with enough confidence and a strong desire to be an entrepreneur. This policy had a direct consequence of rewarding relatively better-off households. Samir Sagar, a staff member in the I/NGO, commented:

Anyone could become an entrepreneur; however, those doing some business [currently or before] on some scale came forward [to receive our support]. They already ran [or had] small businesses in their village. We desired to cover everyone [through our programmes], but those who were already entrepreneurs and those who owned land became the beneficiaries.

Most of the poorest and marginalised households did not have prior business experience due to lack of capital and other factors. As a result, they were considered inexperienced and thus believed to lack the necessary skills and confidence to undertake entrepreneurial initiatives. Above all, the potential beneficiaries needed to contribute a specified amount of capital for entrepreneurship. The project would provide necessary training and specified costs for machinery purchases, and the rest of the costs needed to be borne by the selected beneficiaries themselves. However, the poor and marginalised households, living in precarious situations, lacked the physical and financial assets to do so. On this point, Phul Maya said:

What property can a poor Chepang own! They [our people] have got two goats and one or two cows. If they were rich, they might have a water buffalo. That’s all we have got!...... Others [caste households] were already better off than us. They had plenty of food [in their house]. They had cattle. They are the ‘sāhu’ (money lenders). There were a lot of people who had better conditions than ours. They had a good income and therefore had a good lifestyle: they used to eat good food and wear nice clothes. However, our situation was pathetic at that time [when the earthquake hit]. We were ‘kamjor’ (financially weak or poor). Our husbands used to go out for toiling labour and bring wages and two or four kilos of rice in the evening.

Further, the people who had productive assets–such as buildings, hotels, and vehicles/ jeeps–benefitted remarkably as their services could be rented or hired. Kapadia (2014) observed a similar phenomenon in post-tsunami Sri Lanka where the entrepreneurship programmes reinforced inequitable power relations between poor and wealthy people in the communities because the more affluent households were able to access financial resources conveniently.

### Agency and community contributions

Sarala further explained her story of recovery, returning to farming and selling vegetables. The income was used to fulfil the family's needs. She recalled, “… we started farming again. Now we have our farming back, and we started getting some income [by selling the products in the market]. With that income, we started buying the food and spices needed at home.” Thule Sunar (59), a Dalit male in Dhading District, had a similar experience. He stated, “I ploughed the land [with oxen] and hoed the farmland, and met the needs of the family”. Phul Maya Praja likewise noted, “we were given seeds for off-seasonal vegetable production. We produced tomatoes. After selling them in the market, we sowed bitter gourds and tomatoes.”

Almost every research participant had stories of working in collaboration with other families in the village, providing labour to collectively rebuild community infrastructures damaged by the earthquake. This labour was voluntary and free, and focussed on such things as the repair and rebuilding of drinking water tanks and rural trails. These contributions to the collective could last anywhere from several days to several weeks. Phul Maya Praja’s family volunteered for five days to rebuild a small irrigation system, known locally as *‘kulo’*. Sapana (36), a female Chepang ethnic minority, stated, “We all ‘d﻿āju-bhāi’ and ‘didi-bahini’^3^ (brothers and sisters) in the village got together and rebuilt the damaged drinking water scheme. We donated some money, bought the pipe, brought it and joined it.”

Biru Chepang (53) in Dhading District shared how he worked to help rebuild essential infrastructure. He said, “We rebuilt the drinking water scheme which was damaged by the earthquake. We got some budget from the rural municipality office [for repairing or rebuilding the scheme] and contributed our labour. We worked daily for more than two weeks.”

### *Arko Dasā* (Another Misfortune): the impact of covid-19 pandemic on the post-earthquake recovery journey

The Coronavirus disease 2019 (Covid-19) was another misfortune for the earthquake-affected people. Due to the pandemic, many family members of the disaster-affected households were made redundant in their foreign labour employment, such as in India, Malaysia, and the Gulf countries. In this regard, Phul Maya Praja (41) in the Dhading District said:

Our son had gone ‘bidesh’ [abroad], but he is back home now for he lost his job after Covid. My husband used to earn some money, but he also can’t go out [of home] these days to work. He is compelled to stay at home^4^ [due to the lockdown]…

Further, the disaster survivors working within the country lost their wages and income sources due to the lockdown measures which lasted several months. Dhan Kumari BK (46), a Dalit female in the Dhading District, commented, “We were severely affected by the earthquake. [Following the disaster] our sons in the family were earning well. Our lives were gradually improving but it is destroyed by Corona again”. The situation of Sarala Sunar was devastating. Her family struggles for basic survival. She remarked:

Our ‘chhora’ [son] had a good income in Kathmandu [the capital city]. Life was going well then. But he has no job right now due to Covid. Now we are facing hardships and lots of challenges. It is even difficult to light a fire on the ‘chulo’ (stove).^5^

Similarly, income-generating activities, such as small businesses and micro-entrepreneurship, which played a crucial role for some households in the earthquake recovery process, were also impacted by the pandemic. In this regard, Sapana Chepang (36) shared:

My husband used to make ‘madal’ (a folk drum)^6^ and I used to grow vegetables. Due to the prolonged lockdown, we have not been able to sell even a single madal for months. We were able to resume our business after the earthquake. But these days our business has stopped completely! My husband stays at home all the time [becoming jobless]… There is no market to sell the vegetables [that I grow] either.

Several participants also said that they were struggling to repay the loans taken to run their families and rebuild homes following the earthquake. In this regard, Prem Sunar (29), a male Dalit in the Dhading District, remarked:

I had taken a bank loan by keeping this piece of land as collateral. Now I am being pressurised time and again by the bank staff to repay the loan. What shall I do? I can go nowhere to earn money…

## Discussion

We explored how different aid organisations and government agencies implemented various types of livelihood recovery programmes in the aftermath of the 2015 Nepal Earthquake. These efforts provided twofold benefits to the affected households: addressing the issue of consumption (food security) and providing a means of earning through restoring on-farm and off-farm activities affected by the earthquake.

Our research found that the livelihoods recovery assistance from humanitarian and aid organisations along with the government’s provisions remained crucial in the recovery process; however, several issues and concerns have been observed which will be discussed in the subsequent points. Here, our discussion applies a critical lens to the findings presented in the previous section.

### Livelihood assistance was insufficient and fragmented: how to join the missing links?

In the previous section, we provided a list of livelihood recovery activities implemented by different humanitarian and development organisations and government authorities (Table 3). The livelihood assistance varied: goods/materials support (for example, seeds/seedlings and beehives); animal/livestock support (for example, she-goats and buck/billy goats); tools (for example, a hand-tiller); and capacity building (for example, training events). Similarly, the assistance was either for farm-based livelihood recovery (e.g. commercial vegetable production) or off-farm income-generating activities such as entrepreneurship development and hotel management skills. If we plot these activities in the *livelihood asset pentagon* discussed earlier, they fall under human capital, physical capital, and financial capital (a few). However, the identification and mobilisation of natural capital and social capital in the livelihood recovery programming/process were limited or rare. This result is consistent with those of Nikku et al. (2021) who found that Indigenous strengths and capitals in the Rasuwa District, one of our research locations, went unrecognised in the post-disaster livelihood recovery and reconstruction process in the aftermath of the earthquake. Further, as we discussed in Sect. 5.6, labour outmigration or remittances was a key livelihood strategy for many households. This area remained untouched by the state and non-state actors in our study area. There was an opportunity for these aid agencies to strengthen this sector making the migration process safer or enhancing the skills of youth survivors for increasing their chance of employability.

Furthermore, our findings show that livelihood assistance was limited or incapable of sufficiently raising incomes. As one research participant commented, supporting households with a single goat barely helps improve a family’s lot. Similarly, the income-generating activities that would take multiple years to yield a return are not helpful either. Income-generating activities such as cardamom, coffee, and orange cultivation were not beneficial for survivors as these were enormous time-consuming initiatives. Instead, it would have been preferable to consider income-generating activities that can quickly start generating incomes, rather than waiting years to harness the benefits. Therefore, we argue that there was an opportunity to link the relief *(saving lives)* and recovery *(restoring/rebuilding lives/livelihoods)* related interventions synchronising the activities for short, medium, and long-term impact. At this juncture, the discourse of linking relief to rehabilitation to development (LRRD)^7^ exists.

The discourse of LRRD, the idea of linking humanitarian and development interventions, is a relatively recent phenomenon that was started in the 1980s as scholars and practitioners were seeking to understand the food crisis in Africa (Audet 2015; Mosel and Levine 2014; Otto and Weingartner 2013; Van Dijkhorst 2013). Although it attracted much attention as a discourse in the humanitarian and development field in the 1980s and 1990s intellectual and practical advances were meagre (Mosel and Levine 2014, p. 1). The disaster–development nexus has received renewed attention in the aftermath of the Indian Ocean tsunami in 2004 (see Brusset et al. 2009) and with the emergence of resilience as a salient concept with the promise to link humanitarian and development projects (Mosel and Levine 2014, p. 1). The discussion by Daly et al. (2020) shows how the livelihood activities between relief, rehabilitation, and development stages could be synchronised. Our findings suggest that the livelihood interventions were fragmented or one-off (e.g. distribution of seeds). We argue that systematic and synchronised livelihood activities would be more effective for sustained change as we discuss below.

To elaborate on the LRRD concept for post-disaster recovery context, disaster scholarship (see, for example, Daly et al. 2020; Rasul et al. 2015) suggests that livelihood recovery should be implemented in three overlapping stages: *livelihood provision* (which includes relief-based assistance or short-term measures), *livelihood protection* (which includes early to medium and long-term livelihood recovery), and *livelihood promotion* (which includes transforming livelihoods by reducing the structural vulnerability of the whole livelihood system). Thus, a *systematic* and *sustained* effort is crucial to bring about sustainable livelihood recovery in poor and marginalised disaster-affected households. Daly et al. (2020, p. 12) have shown a model for livelihood recovery illustrating the types of assistance needed at different stages of recovery in relation to housing reconstruction. We build on this discussion in Table 4 by contributing additional scenarios and practical examples. However, our proposal slightly differs from Daly et al. (2020) as we envision the livelihood development (or livelihood promotion) stage as long-term livelihood change or transformation addressing structural marginality, social vulnerability, and equity issues in these communities.

**Table 4:** Examples of systematic and sustained livelihood recovery through the LRRD perspective

Emergency relief assistance *(provision)*	Medium and long-term recovery assistance *(protection)*	Development/transformation facilitation *(promotion)*
Food and non-food relief assistance	*Provision of assets, capital, and stock* to assist in resuming pre-disaster livelihoods or starting a new initiative for marginalised and disadvantaged households	*Targeted provision of assets and capital to scale up*
Cash/voucher assistance for meeting immediate livelihood needs	*Prioritising restoration of vital permanent infrastructure (market, water management facilities, roads, *etc*.)*	*Inclusive employment*
*Provision of cash-for-work for low-skilled reconstruction tasks* such as clearing debris, repairing damaged village footrails, and clearing landslides	Provision of cash-for-work for infrastructure reconstruction, such as irrigation facilities, drinking water reconstruction schemes or school building reconstruction projects	Assistance for livelihood resilience or diversification
Provision for financial services/credit facilities for meeting immediate needs	Provision of seeds/seedlings (considering short, medium and long timeframe to yield or give returns)	Dealing with issues of gender and access to resources, assets or property
Temporary suspension of loan reimbursement by financial institutions from affected households	Provision of financial services/credit facilities for undertaking income-generating activities (on-farm/off-farm)	Effective or just management of natural resources or common-pool resources
Provision of seeds/seedlings that can grow fast in the local environment and basic farming tools for such task; or seeds/seedlings and basic farming tools that are immediately needed due to the ongoing and soon-to-begin sowing or farming time	Vocational skills (basic ones for early recovery and advanced vocational training)	Disaster Risk Reduction (DRR) initiatives
Assistance for temporary shelters for people and livestock	Provision for livestock development support, such as buck/billy goats distribution, livestock rearing training and seedling/samplings for fodder	Address poverty and other social vulnerability issues, including caste and ethnic discrimination
	Forming and building capacity of people’s organisations	Advocacy and policy change on livelihood issues
	*Vocational and small business training including financial literacy training*	Engaging to address market barriers/trade negotiations
		Land reform

The text in italics is adapted from Daly et al. (2020), but it may feature in a different livelihood stage here

### Examining humanitarian objects: mini-tillers in focus

Some disaster-affected households, like Phul Maya in Dhading, were supported with mini-tillers to restore agriculture or food production through the use of modern farming technology (this machine is an example of *physical capital* from the perspective of the sustainable livelihoods framework). However, the participants told us about the challenges of maintaining this technology in rural settings. We build on the insights of Phul Maya to argue that this initiative perpetuates vulnerability in new forms by making people dependent on an external source or agent that undermines resilience as well.

Several scholars have scrutinised humanitarian materials/objects, such as canned meat (Fountain 2014), humanitarian kit (Redfield 2008), personal protective equipment (Pallister-Wilkins 2016), and Plumpy’nut nutritional supplements (Scott-Smith 2013). Their scholarly contributions help us understand the history, political-economy, and biopolitics of humanitarian interventions, using the social lives of material tools and commodities used in the aid industry as points of entry for social analysis. They have also suggested that humanitarian objects are often taken for granted and remain under-studied. In agreement with their argument, our paper focuses on the political economy of the modern technology mini-tillers (also known as hand-tillers or power tillers) distributed to earthquake-affected households for ploughing land. We note that this machine has several benefits. It is more efficient than traditional methods for ploughing the land, for example, using oxen. Women can use a tiller whereas they are not culturally permitted to plough with oxen. However, the power tiller has some drawbacks in the context of rural Nepal.

First of all, people must rely on the market for purchasing and repairing their machines, which benefits dealerships and ultimately the multinational companies that manufacture them. This displaces ownership of livestock, where many raise oxen and bullocks. Sometimes they buy or sell the oxen in the village or the local market, and this contributes to the local economy. Local farmers also sell and buy an ox or oxen to each other or even exchange sometimes with some monetary compensation as agreed. This retains wealth within the community whereas tiller purchases send money to multinational companies. Further, tillers are expensive. They cost more than NPR 50,000.^8^ Tillers also need fuel, and this must be procured in urban market centres, meaning that farmers must commute beyond their immediate locales. Most importantly, the fuel price fluctuates and becomes scarce because Nepal relies on India for fuel. When there was a Nepal-India border blockade five months after the Nepal Earthquake, the fuel supplies to Nepal ceased for several months (BBC 2015).^9^ Some would argue that tillers only need fuel while being used, whereas families need to feed the oxen all year round regardless. There is validity to this argument; however, cash and sometimes fuel are scarce in rural Nepal, while fodder is freely available. Moreover, livestock in rural Nepal are part and parcel of rural life.

Research participants also shared their dissatisfaction that they need to take tillers to distant repair shops. Given the distance to such places and the sheer weight of the machine, this was a great inconvenience. The fact that research participants were unable to repair these machines themselves indicates that the repair/maintenance training was either insufficient and/or that refresher courses are necessary. Further, even if the training was sufficient, the households still need to occasionally procure replacement parts which require money and travel to distant vendors that sell them.

The power-tiller was not a replacement for technology that was lost in the disaster, but a new thing introduced in the aftermath of the earthquake. We argue that any new technology (or new practices) introduced into developing countries—especially in sites of disaster recovery—should be done carefully. Unintended consequences abound. With regard to the power tiller, these aspects are worth reviewing– what is the cost of the new technology, who decides, who benefits (more), how it will be (un-)sustainable, and finally, will it create market dependency or help people to have control over goods/technology? Finally, humanitarian objects like power-tillers are often fetishised, presenting themselves as a magic bullet to solve existing problems; however, food insecurity and hunger in disaster-affected places cannot be solved by a power-tiller alone (see Scott-Smith 2013). Rather, interventions should address the complex and interconnected causes that create social vulnerabilities: unjust distribution of land and other resources between different castes and ethnic groups, lack of irrigation, acute poverty, and lack of access to agriculture extension services. At this juncture, we are making explicit our own position on relief, rehabilitation, and development (LRRD) debates. We are in favour of addressing root causes and bringing about sustained change, not simply just immediate relief (Audet 2015; Mosel and Levine 2014; Rose et al. 2013).

### Market-focused livelihood programs served neoliberal interests

Our findings highlight that livelihood recovery programmes, to a large extent, were market-led or market-oriented. Therefore, disaster survivor dependencies were created on the market. For example, humanitarian agencies distributed hybrid seeds to earthquake-affected households causing them to rely on the market every new season when they need to sow the seeds. Similarly, commercial farming and cash crops remained the focus of assistance in several disaster-affected communities. Further, the aid agencies introduced new technology (which we discussed in the Sect. 6.2) replacing traditional farming methods. These types of assistance also entrench new market dependencies. Therefore, the income generation activities served neoliberal market agendas rather than addressing the unjust distribution of productive assets, such as land, among different caste and ethnic peoples in the village.

We further argue that livelihood activities/assistance that creates too much market or *exogenous* dependency are likely to put people in a vulnerable state by eroding local institutions and systems which certainly impact people’s local reliance, resilience and recovery capabilities (see Bano 2012). In this regard, the discourse on Indigenous resilience also exists which argues to help local people (re-)connect with their natural capital, social institutions, and local systems to improve livelihoods and enhance resilience (see Nikku et al. 2021).

### People’s agency: beyond the narratives of ‘recipients’

The survivors have demonstrated agency, actively contributing to their communities’ recoveries. They utilised official assistance to increase productivity and improve their living conditions. For example, survivors (such as Sarala, Thule and many others) recommenced their farming practices. They also provided days and weeks of free labour to rebuild community infrastructures. This shows that disaster survivors were not merely consumers of humanitarian assistance. Further, the disaster survivors were producers of, and contributors to, relief and recovery efforts. Our findings correlate with other studies that show that the contribution of relief aid was but a small portion of the livelihood needs of local people. For example, in Gautam’s (2019) study, food aid contributed only 20% of the total food needs of families in the Humla District of the Karnali region in Nepal. Similar cases can be found elsewhere. Omata (2017), for example, revealed that people in the Buduburam refugee camp in Ghana relied far more on remittances sent by family members than they did on humanitarian aid. Therefore, in contrast to what may commonly be believed, disaster survivors are not passive recipients of humanitarian assistance.

## Concluding remarks and way forward

Our results highlight that livelihoods recovery aid/assistance provided by the state and non-state actors was a crucial, useful and appreciated disaster recovery strategy in resource-poor settings; however, there can be problems in their implementation that may wittingly or unwittingly follow established vectors of inequality, in turn amplifying them.

We found that livelihood assistance in the study area was predominantly related to human capital, physical capital or financial capital (referring to the asset pentagon of the SL framework). Social capital–for example, sharing of food–was evident during the emergency phase following the earthquake (see Karki et al. 2022b) and during the housing reconstruction process–for example, the mutual exchange of labour (see Karki et al. 2022a; Gautam and Cortés 2021; Panday et al. 2021). However, this important capital was less recognised and underutilised in the livelihood recovery programming/process. We reiterate that social capital–and natural capital too–are also crucial for sustainable livelihood recovery and resilience. We also acknowledge that mobilising these capitals is challenging due to social exclusions (e.g. caste and gender-based discrimination) and the unequal distribution of natural resources between different caste and ethnic groups.

Further, our research revealed that the “replacement” or “restoration” concept (the idea of regaining what was lost or damaged by a disaster) is problematic as it overlooks the pre-disaster vulnerability of poor and marginalised households who experience disproportionate disaster impacts. We also showed how this phenomenon benefits the elites or relatively better-off people in communities. Therefore, this suggests that without pro-poor recovery policies and programmes, pre-disaster inequalities between the haves and have-nots are likely to continue, if not grow, in post-disaster environments.

Congruent with Daly et al. (2020) we see the need to link livelihood relief, rehabilitation and recovery/development. This necessitates the adoption of a holistic livelihood recovery process rather than piecemeal and fragmented livelihood assistance. Further, any new commodity or technology should be carefully assessed to ensure its suitability, viability, and effectiveness in the contexts in which they are to be used so that such initiatives do not create unnecessary dependency on either the market or external actors. At this juncture, we argue that local people’s position and ability to own or have control over the means of production is crucial for resilient livelihoods.

Further, we showed that the earthquake survivors had to go through a second disaster (the Covid-19 pandemic) before fully recovering from the devastating impact of the earthquake. In this regard, it is critical to address the needs of the most marginalised households who were hit hard by two life-threatening disasters, one after another in a relatively short period of time.

Finally, in contrast to what may commonly be believed, disaster survivors are, as we have shown, not passive recipients of humanitarian assistance. Therefore, they should be recognised and encouraged for their willingness and ability to bring about positive changes in the livelihood situation of their families and communities.

## Footnotes

1 The implementing organisation mentioned that it was an unconditional cash support scheme; however it would seem that it was not unconditional as the beneficiaries were asked to utilise the assistance for improving livelihoods (their economic situation) following the earthquake.

2 Pseudonyms have been used to preserve the confidentiality of research participants. However, the surname (family name) has been retained which informs the caste background and ethnic identity (and in some cases, ethnic marginality as well) of an individual.

3 *D﻿āju-bhāi* and *didi-bahini* are literally translated as brothers and sisters respectively, but they are not blood-relatives. Nepali society is very kinship and relation oriented. For example, residents in the village are considered d﻿āju-bhāi and didi-bahini in a general sense. In contrast to the western world, where someone may be referred to as a man (or gentleman) or lady, or by their given name, people in Nepali society are addressed as close relations or relatives, such as *hajurāmā* or *b﻿aje* (grandma), *hajurbā* or *bāje* (grandpa) for any old women or men in the neighbourhood even when not biologically related.

4 “Staying at home” also refers to being jobless.

5 Chulo is a traditional stove made up of stones and mud. However, the expression here is a metaphor which means to cook food.

6 A double-headed traditional folk drum in Nepal.

7 The LRRD debates go by various names in the literature such as the *humanitarian-development nexus*, *disaster–development continuum*, *the double nexus,* and recently the *triple nexus* referring to the interlinkage between humanitarian, development, and peacebuilding activities.

8 According to the USAID funded Feed the Future INGENAES project, the price of the mini-tiller was NPR 50,000 at the time of distribution.

9 The border blockade created a humanitarian crisis affecting people’s everyday life (such as lack of LPG bottles for cooking), commuting to work or travelling, transportation of essential services, and it impacted the supply chain of essential goods and medical supplies. This crisis also halted Nepal’s post-earthquake recovery and reconstruction work (see BBC 2015).
